# Optimizing Pulmonary Rehabilitation in Saudi Arabia: Current Practices, Challenges, and Future Directions

**DOI:** 10.3390/medicina61040673

**Published:** 2025-04-06

**Authors:** Fahad H. Alahmadi

**Affiliations:** Respiratory Therapy Department, College of Medical Rehabilitation Sciences, Taibah University, Medina 41477, Saudi Arabia; fhrahmadi@taibahu.edu.sa

**Keywords:** pulmonary rehabilitation, dyspnea, chronic respiratory disease, asthma, COPD, quality of life, exercise capacity

## Abstract

Chronic respiratory diseases (CRDs) are a significantly major cause of mortality in Saudi Arabia, with their progression frequently involving comorbidities and exacerbations that extend beyond the lungs. This review considers the current state of pulmonary rehabilitation (PR) in Saudi Arabia, this being a well-known non-pharmacological intervention to help control and reduce the burden of CRDs, highlighting the intervention’s availability, multidisciplinary approach, and integration within the healthcare system, as well as examining the diseases’ contribution to overall symptom severity, impairing daily activities and significantly worsening the patient’s quality of life. Although PR is strongly recommended for managing CRDs, its utilization in Saudi Arabia remains limited or unavailable in many regions. Key barriers to PR access include inadequate awareness among healthcare providers and patients, logistical challenges, and an insufficient number of specialized facilities and trained professionals. Expanding PR programs in Saudi Arabia requires addressing geographical barriers, ensuring adequate space, resources, and trained personnel, and raising awareness among healthcare providers through education and training. Integrating PR principles into medical education and offering incentives for specialization can help overcome personnel shortages. Additionally, promoting telerehabilitation can enhance patient compliance and ensure the long-term success of PR programs. These initiatives aim to optimize PR services and improve patient outcomes across the nation.

## 1. Introduction

Chronic respiratory disorders (CRDs) are a significant global health challenge. Asthma and chronic obstructive pulmonary disease (COPD) are the most widely recognized of these disorders and are prevalent among CRD cases, impacting more than 545 million people worldwide, while 4 million deaths were recorded in 2019 [1]. However, less common but still serious CRDs, like bronchiectasis, pulmonary transplants, interstitial lung disease, and occupational lung diseases (such as pneumoconiosis and asbestosis) also pose a considerable health burden. In recent times, post-COVID-19 [2] syndrome has been emerging as a notable contributor to the CRD category, with some patients suffering from long-term respiratory symptoms after recovery from the virus. These diseases can lead to reduced lung function, frequent hospitalizations, and, in severe cases, death. The substantial number of affected individuals highlights the need for more effective treatments, early diagnosis, and preventive measures to reduce the impact of these disorders on public health.

According to the World Health Organization’s (WHO’s) Global Burden of Disease Report, COPD was the sixth-largest cause of death in 1990 and the third-largest cause of death globally by 2020 [1], affecting more than 450,000 individuals in Saudi Arabia [3]. It also became the fifth-largest cause of disability worldwide. According to a Global Burden of Disease survey in North Africa and the Middle East, CRDs made up 2.91% of all disability-adjusted life years (DALYs) [4]. In developing countries, chronic respiratory illnesses are increasing rapidly, causing more health issues, frequent hospital visits, and a significant decline in health-related quality of life (HRQoL) [4]. Pulmonary rehabilitation (PR) is considered a cornerstone in non-pharmacological treatments aimed at reducing the morbidity and mortality associated with CRDs. This evidence-based approach integrates exercise, nutrition, and behavioral and psychosocial interventions to alleviate the extrapulmonary symptoms of CRDs. PR has consistently demonstrated positive outcomes, including reductions in dyspnea, functional limitations, exacerbation frequency, and hospital readmissions, while also enhancing the quality of life [5]. As a result, PR is widely recognized as a key strategy in managing COPD [5]. However, the integration of PR into standard CRD care remains in its early stages, and many patients who could benefit from these programs continue to be overlooked [6].

The American Thoracic Society (ATS) and the European Respiratory Society (ERS) issued a joint statement in 2015, emphasizing the need to bridge the gap between the established benefits of PR and its poor utilization [7]. Despite strong evidence supporting the effectiveness of PR in improving outcomes for patients with chronic respiratory disorders, it remains significantly underused worldwide, including in Saudi Arabia and many other regions [8].

A recent cross-sectional study conducted in Saudi Arabia revealed that only 29% of physicians had referred COPD patients to PR programs, highlighting a significant gap in service delivery [9]. This may be attributed to a lack of knowledge, insufficient healthcare facilities, or improper program implementation [9]. This review study aims to explore the present situation of pulmonary rehabilitation in Saudi Arabia, identifying current gaps in service delivery and barriers to access. It will also offer future paths for the implementation of PR services, to adhere to the international recommendations for CRD management.

## 2. Risk Factors of CRD in Saudi Arabia

There are several risk factors that have contributed to CRD cases in the Kingdom of Saudi Arabia. The predominant contributor to the high incidence of CRDs in Saudi Arabia is tobacco smoking. According to a WHO study in the Eastern Mediterranean region, Saudi Arabia ranks 34th globally and 6th in the WHO in terms of the number of tobacco smokers [10]. Over the past 30 years, several research studies conducted in Saudi Arabia have shown a growing prevalence of smoking, especially among young men and women [11]. As of 2022, 17.8% of the population were current tobacco users, with an estimated 4.8 million individuals aged 15 and older using tobacco daily, cigarettes being the most common form used [12,13]. In Middle Eastern countries, cigarette smoking is a significant risk factor contributing to 35% of esophageal cancer, 30% of lung cancer, and 8% of stomach cancer cases [13].

Moreover, a significant number of individuals in Saudi Arabia have a history of tuberculosis, chronic asthma, and respiratory infections during infancy, all of which are recognized risk factors for CRD [14]. In addition to smoking, other factors such as biomass fuel exposure, dust, harmful gases, and outdoor air pollution also play a major role in the increasing prevalence of CRD in the country [15]. Addressing these risk factors is crucial for implementing effective prevention and management strategies to mitigate the burden of CRD.

## 3. Burden of Chronic Respiratory Disease in Saudi Arabia

In the Middle East and the Gulf Cooperation Council (GCC) region, Saudi Arabia is one of the largest countries, having a population of 35.6 million. Despite the area’s size and significance, COPD remains widely underdiagnosed and undervalued in this region. It is usually only identified after noticeable symptoms appear, and the disease has progressed to a more severe phase [10].

The Global Burden of Disease 2019 provides a detailed epidemiological analysis of diseases and injuries worldwide, from 1990 to 2019. However, data on the prevalence, incidence, morbidity, and mortality of CRDs in Saudi Arabia remain scarce. Between 1990 and 2019, the overall age-standardized prevalence rate of COPD in Saudi Arabia increased by 49% [3]. During this period, the incidence of new COPD cases in terms of age-standardized figures rose by 43%, and COPD-related mortality increased by 48%. Currently, COPD accounts for 1.65% of all-cause deaths in Saudi Arabia and 57% of respiratory-related fatalities overall, including interstitial lung disease and asthma [3]. Additionally, a significant number of patients in Saudi Arabia report a history of conditions like persistent asthma, tuberculosis, and childhood respiratory infections, all of which are known risk factors for the development of COPD [14].

Post-COVID-19 syndrome has further exacerbated the burden of respiratory diseases. This syndrome often results in persistent respiratory symptoms, muscle impairment, and reduced physical activity levels [16]. Saudis rely heavily on personal transportation, which can lead to physical inactivity among both youngsters and adults. Physical inactivity is a significant risk factor for comorbidities and respiratory muscle dysfunction, which can exacerbate respiratory conditions and hinder recovery [17]. These findings highlight the need for urgent public health interventions, including effective smoking cessation programs and initiatives to promote physical activity, which will contribute to lowering the risk of CRD in Saudi Arabia.

## 4. Management of CRD

Chronic respiratory disease management requires a comprehensive approach that integrates pharmacological and non-pharmacological strategies to improve patient outcomes and reduce the disease burden. Pharmacological treatments, including bronchodilators, corticosteroids, and biologic therapies such as omalizumab and mepolizumab, have been effective in controlling inflammation and reducing exacerbation in diseases like asthma and COPD [18]. Despite advances in pharmacological treatments for CRD, a large proportion of patients remain symptomatic and suffer from frequent exacerbation and hospitalization, and the course of these diseases cannot be reversed [19,20]. Interstitial lung diseases comprise a diverse group of chronic, often progressive lung disorders that respond poorly to pharmacotherapy, lead to increasing disability, and contribute to rising healthcare utilization [21]. In addition, CRDs are characterized by extra-pulmonary features, and many patients suffer from comorbidities [21,22]. Physical inactivity is a well-recognized disadvantageous lifestyle factor contributing to disease progression, impaired health status, and decreased exercise capacity, leading to increased mortality. An unhealthy diet, extremes in body weight, and low muscle mass are associated with poor outcomes in patients with COPD and ILD. Many patients also experience difficulties in coping with their disease and have limited knowledge about their condition, as well as poor self-management skills [23].

Non-pharmacological interventions, including PR, exercise therapy, smoking cessation, and proper nutrition, play a crucial role in maintaining lung function and enhancing quality of life [23]. Psychological support, including cognitive behavioral therapy, has also been shown to be essential in mitigating the anxiety and depression associated with CRDs [24]. Advanced drug delivery systems, including nanoparticles and inhaled biologics, offer promising improvements in treatment precision and efficacy [25].

Overall, the combination of innovative therapies, lifestyle modifications, and comprehensive healthcare support is essential for effectively managing chronic respiratory diseases and enhancing patients’ long-term well-being.

## 5. Pulmonary Rehabilitation and Telerehabilitation: An Overview

**Figure 1:**
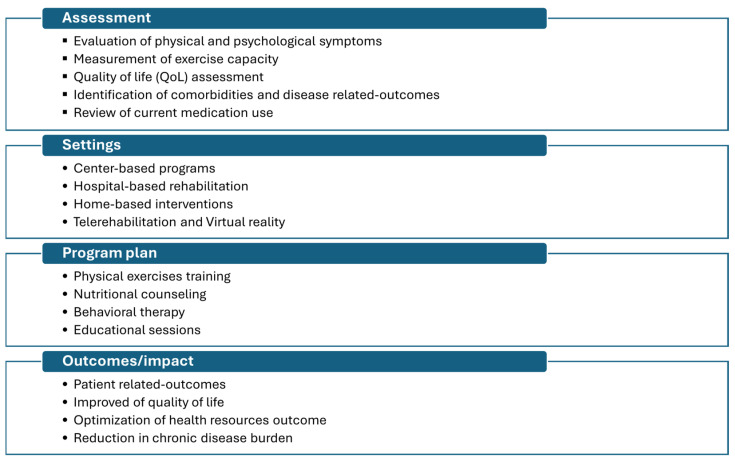
Essential components of pulmonary rehabilitation (source: American Thoracic Society/European Respiratory Society Statement on Pulmonary Rehabilitation) [26].

The pulmonary rehabilitation program is traditionally delivered in hospital/center-based settings. With the advancement of internet access and technology, some developed countries have integrated modern program technology that involves home-/remote-based approaches, known as telerehabilitation. These programs are composed of several interventions other than physical training to achieve multidisciplinary management (as shown in Figure 1) [26].

The positive impact of PR on both patient-related outcomes and healthcare resources is well documented. PR significantly improves muscle strength, endurance, exercise capacity, disease self-management, HRQoL, nutritional status, and physical activity levels [27,28]. Additionally, it reduces the frequency of exacerbations, unscheduled hospital visits, hospitalizations, dyspnea symptoms, leg discomfort, depression, anxiety, and overall healthcare costs in patients with CRDs [29]. Although PR may affect people differently, those who find the intervention helpful are more likely to continue exercising regularly and will attend future PR sessions, further supporting its long-term value [30]. Medicine telerehabilitation has been established to meet patients’ and healthcare providers’ needs and to overcome the barriers to accessing traditional PR facilities. It enables remote service delivery and patient monitoring via a personal computer or smartphone, making it a potentially feasible therapy [31]. Telerehabilitation offers advantages such as accessibility, ease of monitoring, and cost savings for both patients and hospitals [31,32]. It includes exercise videos, instructional materials, and online counseling sessions that can improve patient motivation and compliance with the PR program [31]. It has proven to be a valid and reliable method compared with traditional rehabilitation methods globally [32]. In recent years, Saudi Arabia has embraced digital healthcare advancements to ensure equitable and sustainable services that are compatible with the country’s population growth. The digital health sector in Saudi Arabia developed Seha Virtual Hospital, the region’s first and largest virtual hospital, which is capable of accommodating over 400 patients [33]. This facility is expanding its network of medical services, staff, and facilities. These advancements in digital healthcare provide a unique opportunity to integrate telerehabilitation PR into the evolving healthcare landscape, enabling remote PR services for patients with CRDs. Through tools like video consultations, online exercise programs, and remote monitoring via wearable devices, PR can become more accessible to patients who face barriers to attending traditional in-person programs, such as geographical transportation issues or mobility issues [34,35].

Additionally, digital health technologies can facilitate better patient adherence and engagement through real-time feedback, personalized exercise plans, and educational resources delivered via smartphones or computers. This aligns with the objectives of PR to improve patient outcomes, reduce hospitalizations, and enhance the overall quality of life of CRD patients. Integrating PR services into Saudi Arabia’s robust digital health infrastructure, including initiatives like Seha Virtual Hospital, could ensure a wider reach, cost efficiency, and equitable access to rehabilitation programs across the country [34,35,36].

Although there are promising advantages to telerehabilitation, implementing such services for CRD patients in Saudi Arabia is a challenge. A nationwide study among physiotherapists revealed that technical issues and the associated costs are the primary limitations to integrating telerehabilitation [36]. In addition, communicating with female patients in Saudi Arabia through digital services can raise ethical and legal concerns, as religious and cultural factors may discourage visual interactions with healthcare providers. 

A study exploring cultural factors in digital health interventions found that patients and their families often perceive indirect contact, such as through cameras, as a barrier to effective communication, influenced by cultural views on using cameras during consultations [33]. Additionally, the requirement for women to wear a Hijab, covering their face, head, and body in the presence of non-related men, underscores the importance of cultural sensitivity in healthcare settings [37].

## 6. Pulmonary Rehabilitation’s Current Status and Utilization in Saudi Arabia

In Saudi Arabia, the healthcare system operates on three levels: primary, secondary, and tertiary care [38]. Over the past decade, the number of hospitals in the country has steadily increased, reaching 497 hospitals with 78,000 beds by 2022 [39]. Although the ratio of hospital beds per 1000 people improved in 2022, it remains below the global average. To maintain the current ratio, an estimated 10,200 additional beds will be needed by 2025 [40,41].

Saudi Arabia’s healthcare expenditure in 2020 was around USD 49 billion and is expected to rise to USD 77.1 billion by 2030 [42]. To reduce health care expenditure, it is crucial to find effective strategies for mitigating the chronic disease burden. Respiratory diseases heavily impact the cost and utilization of health care services in Saudi Arabia.

Hospital stays account for half of the total out-of-pocket expenses, followed by the cost of medication therapy. Commonly used treatments such as oxygen therapy and bronchodilators are easily available and are widely promoted [40]. Interestingly, a UK study found that PR was much more cost-effective than bronchodilator therapy when measured by cost per quality-adjusted life year [43].

## 7. Availability and Utilization of PR Services in Saudi Arabia

The first PR program in Saudi Arabia was established at King Abdulaziz Medical City in 2001. Since then, only a small number of PR services have been implemented across the country. Despite the recognized importance of PR in managing CRDs, particularly COPD, access to PR services in Saudi Arabia remains limited [43]. To the best of our knowledge, only a few tertiary care facilities actively provide PR programs, and no data are available on the percentage of patients benefiting from these services. Additionally, even when hospitals have PR initiatives, healthcare providers are often unaware of their existence [44].

Notwithstanding the variety of rehabilitation centers available across Saudi Arabia, there is a lack of PR services within these facilities. While general rehabilitation services may serve patients with a range of conditions such as orthopedic or neurological disorders, PR programs for patients with CRDs remain limited or nonexistent.

The absence of PR services in many centers may be due to several factors, such as insufficient awareness among healthcare providers, a lack of trained personnel specialized in PR, and limited infrastructure to support the multidisciplinary nature of these programs [9,45]. Addressing this gap is crucial, due to the needs of the growing number of CRD patients in Saudi Arabia. Integrating PR into existing rehabilitation centers could significantly enhance their scope and provide much-needed support for patients managing respiratory conditions.

## 8. Pulmonary Rehabilitation Outcomes in Saudi Arabia

Only a few studies have investigated the outcomes of PR on CRDs, particularly in patients with COPD, as illustrated in Table 1. These studies focused on the beneficial effects of PR services for the Saudi population. Notably, only three studies have evaluated the effectiveness of PR programs for respiratory patients in Saudi Arabia, revealing a significant gap in research on PR services in the country. All three studies were conducted at the same medical center in Riyadh [46,47,48], highlighting the limited availability of PR services. Furthermore, these studies appear to have involved the same group of patients, offering valuable insights into the impact of PR programs on healthcare utilization, adherence, and health outcomes over time. However, the lack of studies from multiple centers or broader research efforts underscores the urgent need to expand PR services and research across Saudi Arabia. This gap still exists, despite the variety of rehabilitation centers available in the country.

## 9. Stakeholder Perspectives on Pulmonary Rehabilitation in Saudi Arabia

Several studies have explored stakeholder perspectives on PR in Saudi Arabia, focusing on healthcare providers’ attitudes, knowledge, and perceived barriers [9,44,45]. A 2016 study in the Eastern Province of Saudi Arabia revealed that only 3.2% of healthcare providers were aware of PR programs [44]. The main barriers identified included insufficient hospital capacity, a lack of trained staff, and inadequate funding. Similarly, a 2022 national survey of 980 healthcare providers (nurses, physical therapists, and respiratory therapists) found that the majority recognized PR’s benefits—such as improved exercise capacity and quality of life [45]. However, the study also identified several barriers, including the limited availability of PR centers (i.e., 61%), a lack of trained personnel (i.e., 52%), and restricted authority to refer patients (i.e., 44%).

Physicians’ perspectives align with these findings. A 2022 cross-sectional study involving 502 physicians reported that only 29% had referred COPD patients to PR programs [45]. The primary barriers were limited access to PR centers (69%) and trained staff. Almost 75.5% of physicians preferred home-based PR programs, and 89.2% considered smoking cessation a crucial component of PR. These findings underscore the urgent need to improve PR services in Saudi Arabia by raising awareness, expanding facilities, and training healthcare professionals to deliver PR programs effectively.

Nevertheless, PR can be implemented using the existing medical staff and infrastructure within hospitals. Research also demonstrates that PR can be effectively delivered in a variety of settings, such as homes, community centers, or inpatient facilities. As a result, hospitals in Saudi Arabia can adopt the PR approach that best suits their budget and available resources.

Studies on stakeholder perspectives regarding PR in Saudi Arabia offer important insights, although they also have certain limitations. A primary concern is the use of surveys and self-reported data, which may lead to biases or inaccuracies when assessing healthcare providers’ knowledge, attitudes, and perceived barriers [47]. For example, the studies of Al-Daheir [9,45] included a large number of general practitioners (GPs), who generally lack the authority to refer patients to PR services directly [9]. This raises questions about the relevance of their input, especially on topics like referral practices and the challenges faced. Since GPs are not usually involved in the decision-making or operational aspects of PR referrals, their views might not fully capture the difficulties experienced by specialists, such as pulmonologists, who manage COPD patients more directly. A greater inclusion of pulmonologists among the study subjects could have provided more focused insights into referral practices and the associated barriers. Furthermore, while the study emphasized the role of PR centers in COPD management, the limited number of these centers in Saudi Arabia—only 2–3 centers were operational nationwide at the time when the study was conducted—likely influenced the findings and may have skewed the perceived barriers to access and referral.

Also, the studies largely focus on healthcare providers, with limited attention given to patient perspectives or outcomes. This absence of the patient’s perspective makes it difficult to assess the alignment between provider perceptions and patient needs or experiences, which is critical for designing effective PR programs. Addressing these limitations in future research could provide a more comprehensive and actionable understanding of the barriers to PR implementation in Saudi Arabia.

## 10. Future Directions and Forward Planning

An important first step in expanding pulmonary rehabilitation (PR) programs across hospitals in Saudi Arabia is addressing geographical areas. Barriers to implementation taking place must be thoroughly explored and, if necessary, targeted interventions should be developed to enhance healthcare providers’ understanding and knowledge of PR. A key requirement for launching a rehabilitation program is ensuring adequate space and resources, including the necessary equipment and trained personnel [49].

Therefore, it is advised to raise awareness and improve information about PR in order to implement PR programs and, subsequently, increase the referral rate. This can be accomplished by providing seminars, education, and courses on PR programs and CRD management to physicians and other healthcare providers.

There should be an essential, initial move to help establish PR programs in Saudi hospitals; the obstacles should be further investigated and, if required, targeted to raise healthcare providers’ understanding and knowledge of PR. One of the first requirements for starting a rehabilitation program is having enough room and resources for the initiative, including equipment and skilled personnel [49].

Pulmonary rehabilitation works well, but it takes time and requires a specific infrastructure. Patients and physicians/surgeons must be made aware of its value and practicality if it is to be used extensively. PR needs to be promoted to the community as well. Both undergraduate and graduate training for physicians/surgeons, nurses, physiotherapists, and respiratory therapists should cover the early identification of COPD and PR principles. This calls for a higher degree of cooperation between healthcare providers, community workers, and the public. The personnel deficit may be resolved by offering incentives and training to current employees, thereby encouraging aspiring medical professionals to specialize in respiratory conditions [50]. Therefore, it is essential to persuade Saudi Arabia’s healthcare authorities that PR is a critical component in the treatment of COPD and other chronic respiratory conditions.

Telerehabilitation, which utilizes educational materials, exercise videos, and online counseling, can help patients stay motivated during PR programs, improving compliance and ensuring long-term success. Globally, telerehabilitation has proven to be as effective and reliable as traditional in-person rehabilitation programs.

## 11. Conclusions

Although the effectiveness of PR programs as a nonpharmacological intervention for long-term respiratory disorders is well known, the availability of PR services in Saudi Arabia is suboptimal. Notwithstanding the variety of rehabilitation services available, Saudi Arabia still requires them, and one of the greatest difficulties is in finding the multi-professional teams and specialized knowledge required to launch and manage such initiatives.

## Figures and Tables

**Table 1:** Pulmonary rehabilitation studies on the different aspects covered in Saudi Arabia.

Author and Year	Objective	Study Design	Participants’ Characteristics	Outcomes
M.S. Al Moamary, 2012 [48]	To evaluate PR on health care utilization and to find factors that predict good adherence to the PR program	Retrospective study (Data were analyzed for 12 months pre- and post-pulmonary rehabilitation)	51 patients (15 males, 36 females; mean age 57.2 years) with interstitial lung diseases, bronchiectasis, asthma, and scoliosis	-functional exercise capacity 6-minute walking distance (6MWD) improved by an average of 113 m across all patients (from 226 m to 339 m). -Healthcare utilization patterns (ER visits reduced from 2.5 to 0.9 visits per patient, outpatient clinic visits decreased from 5.3 to 2.8 visits, OCS reduced from 117 mg to 33 mg, and antibiotic use dropped from 2.0 to 0.9 courses per patient, which may help to predict the level of adherence to the PR program) -Adherent patients showed better outcomes than non-adherent ones across all metrics
M.S. Al Moamary, 2010 [47]	To assess the impact of pulmonary rehabilitation (PR) on health care utilization and functional outcomes in COPD patients, while identifying barriers to adherence	Retrospective study	62 patients who were diagnosed with COPD were referred to PR—50 patients enrolled; 27 (54%) were adherent and 23 (46%) non-adherent. Patients had a mean age of 66 years and a mean FEV1 of 49%	-Adherent Group: Reduced emergency department visits (2.0 to 0.8 per year), hospital stays (2.2 to 0.6 days), outpatient visits (5.3 to 2.6 per year), short-acting bronchodilator use (9.9 to 4.9), prednisone dose (379 mg to 260 mg), and antibiotic use (3.2 to 1.5 courses) -Non-Adherent Group: Increased prednisone and antibiotic use. Improvements in 6-minute walking distance (218 to 339 m) -Barriers to Adherence: 7 were hospitalized (30.4%), 3 had transportation issues (13.0%), 2 reported program intolerance (8.6%), and 11 were unspecified (47.8%)
M.S. Al Moamary, 2008 [46]	To present the experience of implementing the first pulmonary rehabilitation (PR) program in Saudi Arabia and assess its impact	Prospective cohort study over 30 months	121 patients referred to PR: 89 (73.6%) attended, and 32 (26.4%) did not. Of the attendees, 51 (57.3%) adhered, and 38 (42.7%) did not complete the program. Diagnoses included COPD, bronchiectasis, chronic asthma, interstitial lung disease, and kyphoscoliosis	Significant improvements in adherent patients: Exercise Capacity: 6MWD increased from 216 ± 110 m to 544 ± 269 m; treadmill, arm ergometer, and bicycle performance significantly improved Non-Adherence Causes: Transportation issues (34.2%), hospital admission (23.7%), and unspecified reasons (42.1%) Program Feasibility: Demonstrated improvements in exercise performance and physical fitness
